# Cationic Dye Removal Using Novel Magnetic/Activated Charcoal/β-Cyclodextrin/Alginate Polymer Nanocomposite

**DOI:** 10.3390/nano10010170

**Published:** 2020-01-18

**Authors:** Sushma Yadav, Anupama Asthana, Rupa Chakraborty, Bhawana Jain, Ajaya Kumar Singh, Sónia A. C. Carabineiro, Md. Abu Bin Hasan Susan

**Affiliations:** 1 Department of Chemistry, Govt. V.Y.T. PG Autonomous College, Durg 491001, India; sushmabhilai80@gmail.com (S.Y.); anurakeshbhilai@gmail.com (A.A.); roopachakraborty1991@gmail.com (R.C.); bhawanajain123@gmail.com (B.J.); 2 Centro de QuímicaEstrutural, Instituto Superior Técnico, Universidade de Lisboa, Av. RoviscoPais 1, 1049-001 Lisboa, Portugal; sonia.carabineiro@tecnico.ulisboa.pt; 3 Department of Chemistry, University of Dhaka, Dhaka 1000, Bangladesh; susan@du.ac.bd

**Keywords:** nanocomposites, polymer beads, cationic dye, adsorption, isotherm, kinetics

## Abstract

New magnetic iron oxide (Fe_3_O_4_)/activated charcoal (AC)/β-cyclodextrin (CD)/sodium alginate (Alg) polymer nanocomposite materials were prepared by direct mixing of the polymer matrix with the nanofillers. The obtained materials were utilized as nano-adsorbents for the elimination of methylene blue (MB), a hazardous water-soluble cationic dye, from aqueous solutions, and showed excellent regeneration capacity. The formation of the nanocomposites was followed by high-resolution transmission electron microscopy (HRTEM), scanning electron microscopy (SEM) equipped with energy dispersive X-ray spectrometry (EDX), Fourier-transform infrared spectroscopy (FTIR), vibrating sample magnetometer (VSM), X-ray diffraction (XRD) and adsorption of N_2_ at −196 °C. The rate of adsorption was investigated varying several factors, namely contact time, pH, amount of adsorbent and MB concentration on the adsorption process. Studies dealing with equilibrium and kinetics were carried out in batch conditions. The obtained results indicated that the removal rate of MB was 99.53% in 90 min. Langmuir’s isotherm fitted better to the equilibrium data of MB. Fe_3_O_4_/AC/CD/Alg polymer beads shows amazing adsorption capacities in the elimination of cationic dyes (2.079 mg/g for polymer gel beads and 10.63 mg g^−1^ for dry powder beads), in comparison to other adsorbent materials. The obtained adsorbent is spherical with hydrophobic cross-linked surface properties that enable an easy recovery without any significant weight loss of in the adsorbent used.

## 1. Introduction

Several contaminants with organic and inorganic nature end up often in water sources. Some of them are dyes, which have harmful effects on the environment, animals and humans. Even when present in small amounts, dye compounds discharged into water sources can affect the aquatic life. Synthetic dyes are often utilized for different purposes, like food coloring, textiles, paper, leather, cosmetics, medicines, inks for printing, paints, lacquers, soaps and lubricants [1]. Therefore, these dyes enter the water streams, heading to the sea, avoiding domestic and industrial effluents. This creates a large health risk for aquatic and human life, as usually those organic compounds and their products have mutagenic or carcinogenic effects on humans, even at low concentrations [2]. Textile dyes are strongly colored and non-biodegradable [3,4]. Around 2% of the textile industry dyes go away as effluent. Therefore, the estimated dyes concentration in wastewater is about 10–200 ppm. However, such low concentration can still be visible in the environment. Several chemical, physical and microbial methods, including chemical oxidation, advanced oxidation, membrane technologies, coagulation-flocculation, electrochemical techniques biological degradation, fluorometric determination [5] and also adsorption, were used for elimination of these compounds from wastewater. Adsorption, in particular, is highly efficient and non-toxic, being largely used in the elimination of dyes from wastewater [6,7]. Several types of materials, namely activated carbons [8], functionalized graphene oxide [9], polymers like polyvinyl alcohol (PVA) [10], kappa-carrageenan/PVA nanocomposite hydrogels [11], aerogels from activated carbon [12],wastes from agriculture [13], red mud [14] and zeolites [15], were used as adsorbents for methylene blue (MB) removal from industrial sewage. More recently, non-toxic, highly sensitive, low cost, effective, renewable and eco-friendly materials like three-dimensional, vertical platelets of ZnO carriers [16], monolithic scaffolds [17], having high adsorption capacity and good mechanical and thermal stability are a top research topic in purification of wastewater. Han et al. [18] used encapsulating TiO_2_ into a polyacrylonitrile composite coated with polyvinyl alcohol to remove MB dye. Balkizetal. [19] used quasi cryogel beads based on graphene oxide-alginate for the removal of the same compound. Raghunath et al. [20] used a polymer nanocomposite with proline for the elimination of several textile dyes from an industrial effluent. Ge et al. [21] removed MB using a magadiite–magnetite nanocomposite. Nair et al. [22] used composites of chitosan and lignin for dyes and metal ions adsorption, while Manikandan et al. [23] used physically/chemically modified *Ceibapentandra* seeds. Natural polymeric materials are presently gaining more attraction, being used as adsorbents in wastewater treatment, as they were found to be non-toxic and biodegradable [24]. Activated charcoal has also been extensively used as a common “universal” adsorbent, since it is a simple, safe, high surface area and high adsorption capacity material [25].

β-Cyclodextrin (often named as β-CD) is an oligosaccharide with cyclic structure, containing seven glucopyranose units connected by α-(1,4) linkages. β-CD has an inner cavity with hydrophobic nature, while the exterior is hydrophilic [26]. Such structure ensures an outstanding ability to create complexes with organics in solution [27].

The β-cyclodextrin can form complexes with several molecules, like aromatic compounds by means of intermolecular weak forces. Particularly, β-CD based polymers are quite effective in the elimination of reactive and disperse dyes from water solutions [28]. Hence, the addition of β-CD to composites can improve their adsorption capacity [29].

Functionalized polymer nanocomposites attracted considerable interest due to their physical and chemical features, availability, easy separation and different reactive groups on the backbone chain [30,31]. Alg is biocompatible, non-toxic and contains several carboxyl and hydroxyl moieties, is able to crosslink with different polyvalent ions (like Ba^2+^, Ca^2+^ and Fe^3+^) and is easily adsorbs heavy metals [32], dyes [33] and antibiotics [34,35]. At the present, several researchers want to obtain polymer containing nanocomposites, combining polymers and magnetic particles, which have the advantage of both types of materials, increasing stability and strength [36].

In recent years, the polymer nanocomposites have been acquiring a great interest due to the not so high costs of production and good removal efficiency of some dyes [37,38]. Hydrogel is a porous polymer with a three-dimension network, and its unique arrangement eases the flow of solutes to the inside. Moreover, hydrogel beads have many functional groups in the surface area, which can easily adsorb cationic dyes from wastewater [39].

The present work aims to continue to explore the advantages of both polymeric and nanoparticle-based materials. In particular, our aim is to synthesize and characterize magnetic (Fe_3_O_4_)/activated charcoal (AC)/β-cyclodextrin (CD)/sodium alginate (Alg) polymer composite gel beads. The sodium alginate-based material has high mechanical properties and high MB adsorption capacity, excellent regeneration ability and good separation properties [40].

The MB adsorption capacities of Fe_3_O_4_/AC/CD/Alg composited were tested by varying the kinetics of adsorption, isotherms, pH, restoration and recycling of the materials. Most of the research in adsorptive removal of organics/inorganics has been focused on improving the performance of the materials and enhancing removal rates, [41,42] whereas, less work is carried out on the storage, disposal and reuse of adsorbate-loaded adsorbents, which we present in this paper. Moreover, we used dry polymer beads. We found out that after drying the beads, the resultant powdered composite was easier to handle and store for later use, apart from showing an outstanding adsorption capacity. The material is also biocompatible, non-toxic, can efficiently be utilized for the adsorption of cationic dyes and is easy to regenerate.

## 2. Experimental

### 2.1. Reagents

(C_6_H_7_NaO_6_)*_n_* (sodium alginate), FeCl_3_·6H_2_O (ferric chloride), FeCl_2_·4H_2_O (ferrous chloride), CaCl_2_ (calcium chloride), NH_3_ (ammonia) solution, NaOH (sodium hydroxide) and HCl (hydrochloric acid) were obtained from Merck (Mumbai, India). In the experiment, 0.1 M solutions of NaOH and HCl were used for pH adjustment. Activated charcoal, β-cyclodextrin, MB and ethanol were received from HIMEDIA (Mumbai, India). The structure of MB is illustrated in Figure S1A Deionized water was used throughout the process.

### 2.2. Preparation of Fe_3_O_4_-AC Nanocomposites

Fe_3_O_4_-AC nanocomposites were prepared according to the literature [43], with slight modifications. Briefly, 1.253 g of FeCl_2_·4H_2_O and 3.462 g of FeCl_3_·6H_2_O were separately dissolved in 50 mL of distilled water, mixed together under vigorously stirring until a transparent solution was obtained. The mixed solution was precipitated by addition of 20 mL of ammonia solution (25%, v/v), dropwise, with strong stirring. A dark precipitate was immediately obtained, which was heated to 80 °C for 30 min. The formed black nanoparticles were removed with the help of an external magnetic field and three times washed with double distilled water and ethanol, heated at 250 °C in air for 2 h. The finely prepared 1.0 g Fe_3_O_4_ nanoparticles were dispersed in 10 mL double distilled water and then 1.0 g AC was added to the solution (1:1) and continuously stirred for 2 h. The liquid was removed from the resulting black slurry by decantation and filtration, and the solid was washed with double distilled water several times. The obtained precipitate was dried in an oven at 90 °C for 12 h and 500 °C for 2 h in a muffle furnace.

### 2.3. Preparation of β-CD Coated Fe_3_O_4_-AC Nanocomposite

The obtained 1.0 g Fe_3_O_4_-AC nanoparticles were mixed with 10 mL double distilled water to obtain a colloidal solution that was mixed with 1.0 g β-CD under intense stirring at 40 °C. The formed nanoparticles were collected by applying an external magnetic field. The particles were washed with double distilled water several times and dried in an oven at 90 °C for 24 h.

### 2.4. Preparation of Fe_3_O_4_/AC/CD/Alg Polymer Composite Gel Beads

2.0 g of powder sodium alginate were dissolved into 75 mL double distilled water with strong stirring until a viscous solution was obtained. Then 1.5 g of Fe_3_O_4_/AC/CD nanoparticles were well mixed with the solution. 2% CaCl_2_ solution was added to the gel beads with a syringe with non-stop stirring (300 rpm) and stored overnight to complete gelation and form stable beads. The latter were repeatedly washed with double distilled water and kept in an aqueous medium for later use. A scheme outlining all the steps involved in the synthesis of Fe_3_O_4_/AC, Fe_3_O_4_/AC/CD and Fe_3_O_4_/AC/CD/Alg nanocomposites, from preparation to analysis, is shown in Figure S1B.

### 2.5. Analytical Procedures

N_2_ adsorption and desorption analysis of Fe_3_O_4_/AC/CD/Alg polymer composite gel beads were carried out at −196 °C in a BELSORP-miniII, BEL, Japan apparatus. The samples were heated at 120 °C for 3 h in N_2_ atmosphere, to eliminate any water or gas adsorbed on the surface, on a pretreatment unit (BELPREP-flowII, BEL, Japan). The surface area was determined by the Brunauer–Emmett–Teller (BET) method with data from the N_2_ adsorption and desorption isotherms. The total pore volume (*V_tot_*) was determined from the quantity of N_2_ adsorbed at a *P/P*_0_ = 0.99. The pore size distribution was obtained by the Barrett–Joyner–Halenda (BJH) method. The prepared Fe_3_O_4_-AC, Fe_3_O_4_/AC/CD and Fe_3_O_4_/AC/CD/Alg polymer composite gel beads were characterized by X-ray diffraction (XRD) using a Philips PW3064/60 (Amsterdam, Netherlands). Morphology was studied on a JEOL (Model JSM-6390, Tokyo, Japan) analytical scanning electron microscope (SEM). A Fourier transform infrared spectrometer (FTIR; Japanese shift—JIS, Tokyo, Japan) was utilized to record the infrared spectra of Fe_3_O_4_-AC, Fe_3_O_4_/(AC)/β-(CD) and Fe_3_O_4_/AC/CD/Alg polymer composite gel beads in the range of 400–4000 cm^−1^, with KBr pellets. The microstructure of the prepared nanocomposite beads was analyzed by energy dispersive X-ray spectrometry (EDX; Oxford instruments, Abingdon, UK). The magnetic properties were recorded for the Fe_3_O_4_/AC/CD/Alg polymer composite gel beads with a vibrating sample magnetometer (VSM Lakeshore, model 7410, Carson, CA, USA). High resolution transmission electron microscopy (HR-TEM) images were obtained in a JEOL (Model JEM 2100, Tokyo, Japan).

### 2.6. Adsorption Experiments

The MB adsorption experiments were conducted in batch conditions using the Fe_3_O_4_/AC/CD/Alg polymer composite gel beads. A standard MB stock solution was used and diluted with deionized water in order to obtain different concentrations. The obtained MB solutions was kept in a flask with fixed volume (10 mL of 5 ppm) and the Fe_3_O_4_/AC/CD/Alg polymer gel beads were added (in different amounts) to the MB solutions. The flask was placed in a shaker (150 rpm) for 90 min, at pH 6, in an orbital incubator shaker, at room temperatures. The beads were removed with the help of an external magnetic field, when the experiment was over. The upper layer liquid was analyzed by UV-Vis (Systronics UV-Vis spectrophometer-117, Mumbai, India), at a wavelength of 664 nm, after a certain time. The synthesis of Fe_3_O_4_/AC/CD/Alg polymer composite gel beads and image of gel beads being attracted by the external magnetic field are shown in Figure S2A–D. The removal (*R*, %) was calculated from Equation (1) and adsorption capacity (*q_e_*, mg L^−1^) from Equation (2), as follows:

(1) $$R\left(\%\right)=\frac{C_{o}-C_{e}}{C_{o}}\times 100,$$

(2) $$q_{e}=\left(\frac{C_{o}-C_{e}}{m}\right)\times V,$$

(3) $$q_{t}=\left(\frac{C_{o}-C_{t}}{m}\right)\times V,$$

where *R*, $q_{e}$ and *q_t_* (kinetic adsorption capacity of MB dye) were calculated by Equations (1)–(3), respectively [44], where *C_o_* and *C_e_* are the initial and equilibrium concentrations of MB (mg L^−1^), respectively, *C_t_* is the concentration of MB at a given time *t*, *m* is the adsorbent mass (g) and *V* is the dye solution volume (mL). The equilibrium adsorptions of MB dye from aqueous solutions were investigated using the Langmuir and Freundlich models for isotherms. Furthermore, the kinetics of MB dye adsorption was examined using pseudo-first order and pseudo-second order kinetics. The Fe_3_O_4_/AC/CD/Alg beads were desorbed using 0.1 mol L^−1^HCl solutions after adsorption of MB until saturation. The flask containing 0.1 mol L^−1^ HCl and the saturated Fe_3_O_4_/AC/CD/Alg beads was placed in an orbital incubator shaker at room temperature, for 1 h, to full desorption. Subsequently, beads were filtered and three times washed until neutrality was reached before the following adsorption experiment.

### 2.7. Desorption and Reusability

Fe_3_O_4_/AC/CD/Alg polymer gel beads (0.2 g) were placed in a conical flask containing MB solution (10 mL, 5 mg/L) and the mixture was shaken in an orbital incubator shaker for 90 min at room temperature. The mixture was separated with a magnet and the final dye concentration was found. The Fe_3_O_4_/AC/CD/Alg polymer gel beads were recycled by washing with 0.1 N HCl and water under constant stirring for three times, respectively. Then the gel beads were added into another MB solution in order to begin a new adsorption. The recyclability of the material was followed up to five consecutive adsorption–desorption cycles.

## 3. Results and Discussion

### 3.1. Characterization of the Nanocomposites

#### 3.1.1. Transmission Electron Microscopy (TEM)

**Figure 1:**
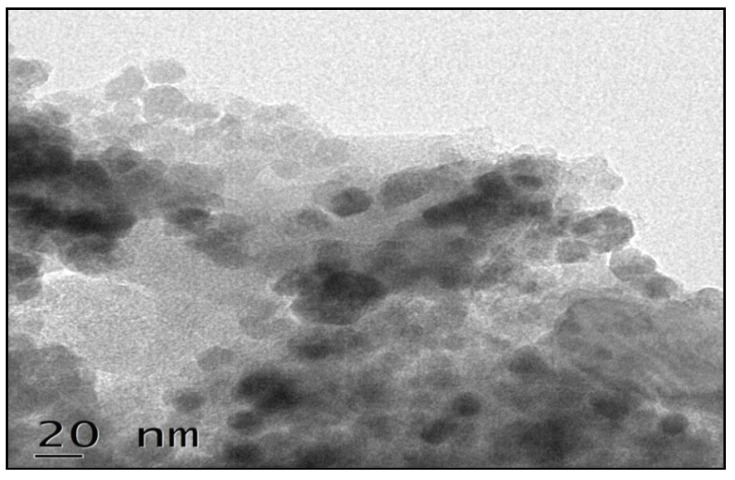
TEM image of Fe_3_O_4_/AC/CD/Alg polymer beads.

High resolution Transmission Electron Microscopy (HR-TEM) was used to investigate the Fe_3_O_4_/AC/CD/Alg in more detail, and the results were shown in Figure 1. TEM showed the nano-sized particles in the synthesized material. The Fe_3_O_4_/AC/CD/Alg is a highly mesoporous material with particle sizes around 20 nm, and grain sizes around 2–15 nm. This decrease in size is mainly due to β-CD [43].

#### 3.1.2. Scanning Electron Microscopy with Energy Dispersive X-ray Spectrometry (SEM-EDX)

**Figure 2:**
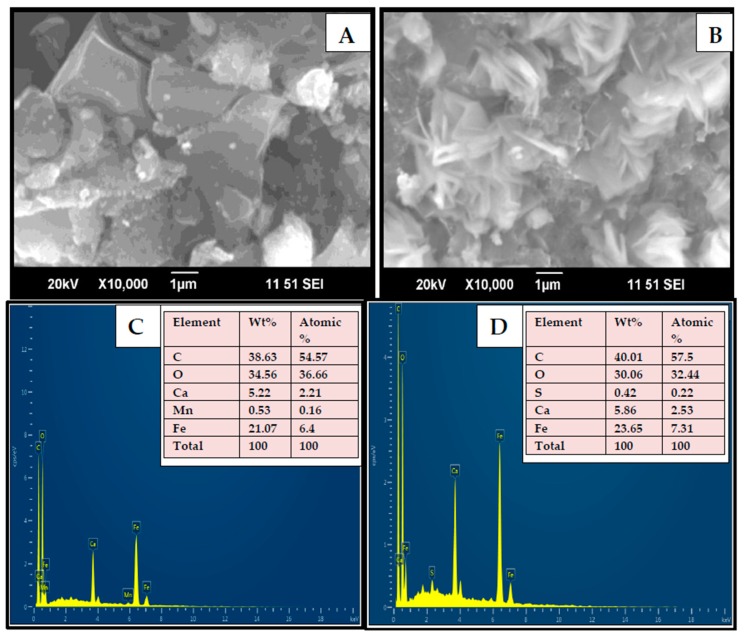
SEM-Energy Dispersive X-ray Spectrometry (EDX) images of Fe_3_O_4_/AC/CD/Alg polymer beads before (**A**) and after (**B**) adsorption. Elemental analysis of Fe_3_O_4_/AC/CD/Alg polymer beads before (**C**) and after (**D**) adsorption.

Figure 2 shows Scanning Electron Microscopy (SEM) results of Fe_3_O_4_/AC/CD/Alg polymer beads before (A) and after adsorption (B), indicating that the surface of Fe_3_O_4_/AC/CD/Alg polymer beads had rough porous structure and large surface area. A 1000× magnification was used. Figure 2A showed that the surface of the untreated beads were uneven, rough and contained pores, which provided suitable binding sites for MB molecules. After adsorption, the dye molecules lead to the coverage of most of the available pores present in polymer beads, causing the surface of the beads to become saturated and much rougher crystallite surfaces were seen, as in Figure 2B. Fe_3_O_4_/AC/CD/Alg showed irregular structure and large surface area, which improved the material adsorption capacity. The elemental analysis of the Fe_3_O_4_/AC/CD/Alg polymer beads are shown in Figure 2C. After adsorption, carbon, oxygen, calcium, iron and sulphur elements were found in MB adsorbed polymer beads as shown in Figure 2D. This indicated a successful adsorption of the dye (from which sulphur also comes from) and the well-distributed adsorption active sites. EDX results of Figure 2D confirmed the elemental analysis findings (insert Table in Figure 2C,D). Before adsorption, the presence of Mn(II) comes from the chemical composition of activated charcoal (as reported in [45]). This very small amount of Mn(II) was adsorbed by the nanocomposite and thus is no longer present after adsorption.

#### 3.1.3. X-ray Diffraction (XRD)

**Figure 3:**
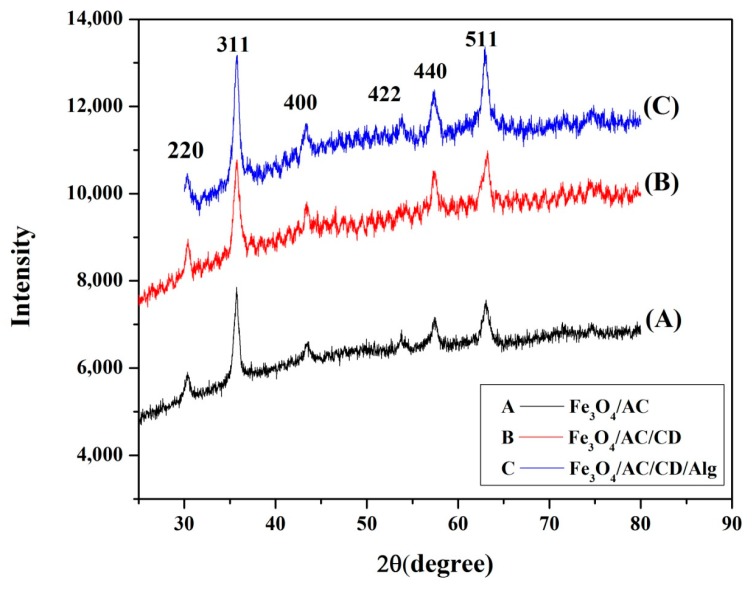
X-ray diffractograms of Fe_3_O_4_/AC (**A**), Fe_3_O_4_/AC/CD (**B**) and Fe_3_O_4_/AC/CD/Alg (**C**).

The structural and crystalline phases of the magnetic nanocomposites were confirmed by XRD. Figure 3 shows the XRD diffractograms of the Fe_3_O_4_/AC (A), Fe_3_O_4_/AC/CD (B) and Fe_3_O_4_/AC/CD/Alg (C) polymer nanocomposite beads, showing the presence of magnetic Fe_3_O_4_ particles, which can be utilized for magnetic separation (Figure 3B). The strong diffraction peaks were indexed to (220), (311), (400), (422), (511) and (440) for all the three samples. The planes appeared at 2θ = 30.1°, 35.73°, 43.45°, 53.22°, 57.40° and 63.07°, respectively. These results matched well with the face-centered cubic (fcc) structure of Fe_3_O_4_ standard XRD data and thus Fe_3_O_4_/AC/CD/Alg could have good magnetic performance for magnetic separation. The wide diffraction peaks are indicators of small sized ultra-fine nanoparticles [46]. The average crystal size (D) of Fe_3_O_4_/AC, Fe_3_O_4_/AC/CD and Fe_3_O_4_/AC/CD/Alg were determined by Scherrer’s equation [47].

(4) $$D=\frac{\left(0.9\lambda \right)}{\beta COS\left(\theta \right)}.$$

In the present case, the mean crystallite size of the Fe_3_O_4_/AC, Fe_3_O_4_/AC/CD and Fe_3_O_4_/AC/CD/Alg (C) nanocomposite were 11.04, 2.71 and 5.63 nm, respectively, as shown in Table 1.

#### 3.1.4. Fourier Transform Infrared (FTIR)

**Figure 4:**
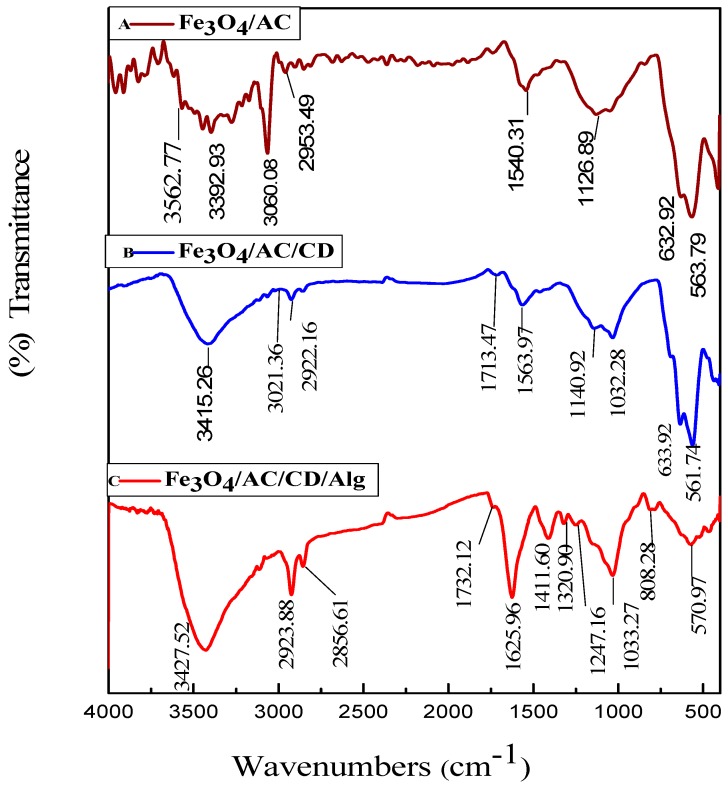
FTIR patterns of Fe_3_O_4_/AC (**A**), Fe_3_O_4_/AC/CD (**B**) and Fe_3_O_4_/AC/CD/Alg (**C**).

The adsorption capacity of an adsorbent is related with its molecular structure and functional groups. For the identification of the functional groups, the Fe_3_O_4_-AC, Fe_3_O_4_/AC/CD and Fe_3_O_4_/AC/CD/Alg polymer composite beads were analyzed by FTIR and results are in Figure 4. For Fe_3_O_4_-AC, the bands at 3562.77, 3392.93, 3060.08, 2953.49, 1540.31, 1126.89, 632.92 and 563.79 cm^−1^ were observed in Figure 4A. The bands at 632.92 and 563.79 cm^−1^ were ascribed to Fe–O vibration modes, just like for Fe_3_O_4_/AC/CD and Fe_3_O_4_/AC/CD/Alg. The bands at 3562.77 and 3392.93 cm^−1^ were due to –OH and –NH_2_ stretching vibrations. The band at 3060.08 cm^−1^ was from the (=C–H) stretching vibration. The band at 2953.49 cm^−1^ was assigned to the antisymmetric stretching vibrations of the methylene group. The peaks for the C–N stretching vibration were found at 1126.89 cm^−1^. For Fe_3_O_4_/AC/CD, bands at 3415.26, 3021.36, 1713.47, 1563.97, 1140.92, 1032.28, 633.92 and 561.74 cm^−1^ were observed (Figure 4B). The band at 3392.93 cm^−1^ was ascribed to the –N–H stretching in Fe_3_O_4_/AC, which was shifted to 3415.26 cm^−1^ in case of Fe_3_O_4_/AC/CD, due to the formation of H-bonds between the –N–H group in Fe_3_O_4_/AC and the –OH groups in β-cyclodextrin polymer [48]. The band at 3021.36 cm^−1^ came from (=C–H) stretching vibration. The peak at 1713.47 cm^−1^ was related with the stretching vibrations of the –C=O carbonyl groups and 1563.97 cm^−1^ was from the N–H bending of –NH_2_. Peaks at 1140.92, and 1032.28 cm^−1^ (stretching of O–C–O of ether groups and stretching of –C–O of alcoholic groups) related to the glycosidic (C–O–C) antisymmetric vibrations and ʋ(C–C/C–O) coupled stretching vibration. Therefore, it could be concluded that Fe_3_O_4_/AC/CD was present [43]. For Fe_3_O_4_/AC/CD/Alg (Figure 4C), bands at 3427.52, 2923.88, 2856.61, 1732.12, 1625.96, 1411.60, 1320.90, 1247.16, 1033.27, 808.28 and 570.97 cm^−1^ were found. The bands at 570.97 cm^−1^ (characteristic peak of Fe_3_O_4_) and at 3427.52, 2923.88, 2856.61, 1625.96, 1411.60 and 1033.27 cm^−1^ (for sodium alginate) were ascribed to –OH (stretching vibration), –CH_2_ (stretching) from CH_3_,–COO^−^(asymmetric stretching), –CH_2_ (bending) and C–O–C (stretching) vibrations [49,50], respectively. The peaks appearing at 1732.12 cm^−1^ were related to the carboxylic groups –C–O stretching vibrations. The absorption bands at 1320.90, 1247.16 and 808.28 cm^−1^ could be ascribed to the C–OH stretching vibrations, C–O–C stretching vibrations and C–H bending vibrations, respectively.

#### 3.1.5. Vibrating Sample Magnetometry (VSM)

**Figure 5:**
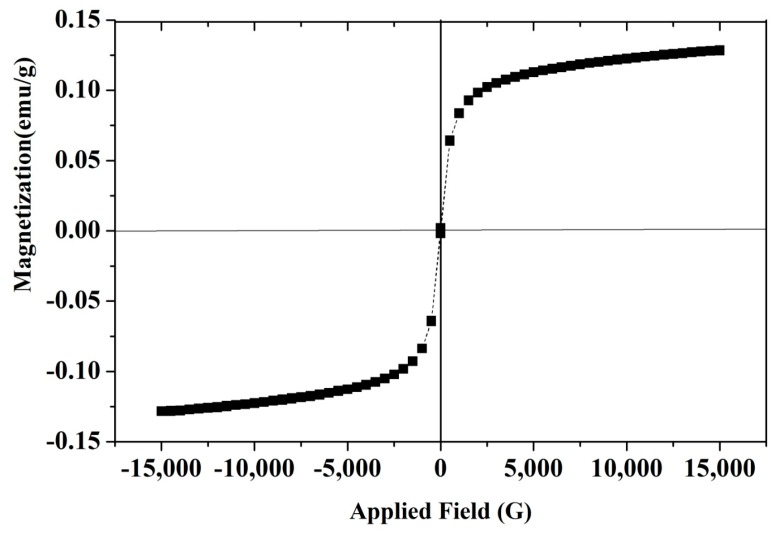
Magnetization curves of Fe_3_O_4_/AC/CD/Alg polymer composite gel beads at room temperature.

To determine the magnetic properties of the obtained Fe_3_O_4_/AC/CD/Alg nanocomposite, the magnetic-hysteresis (MH) loops were recorded at room temperature. Results can be found in Figure 5. The Fe_3_O_4_/AC/CD/Alg nanocomposite exhibited hysteresis, which confirmed the usual ferromagnetic behavior of nanoparticles with negligible coercivities and moderate saturation magnetization. From MH loops, the saturation magnetization value (M_S_) of Fe_3_O_4_/AC/CD/Alg was found to be 0.12849 emu g^−1^. Since the composite was composed of a mixture of three non-magnetic materials, viz. activated charcoal, β-(CD) and sodium alginate, the M_S_ obtained for Fe_3_O_4_/AC/CD/Alg had a lower value. The results were in conformity with those reported for the β-(CD) coated Fe_3_O_4_/carbon nanocomposite in literature, for which the M_S_ was 1.2 emu g^−1^ [43]. The adsorbent could be easily separated using a magnet, showing easier operation due to the sensitive magnetic response.

#### 3.1.6. Textural Analysis

**Figure 6:**
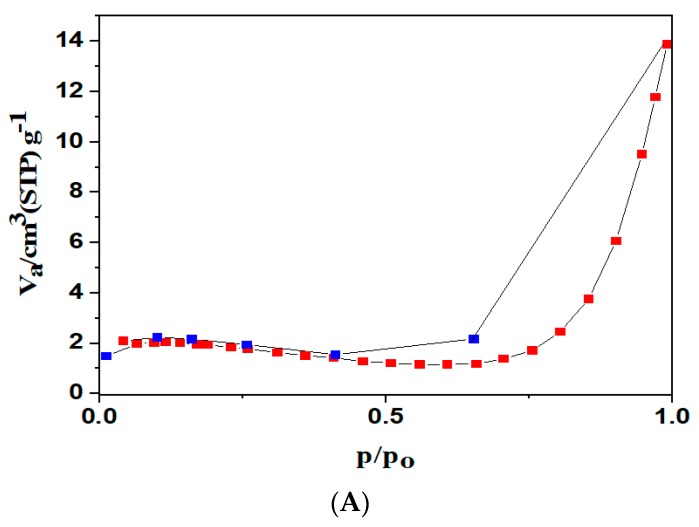

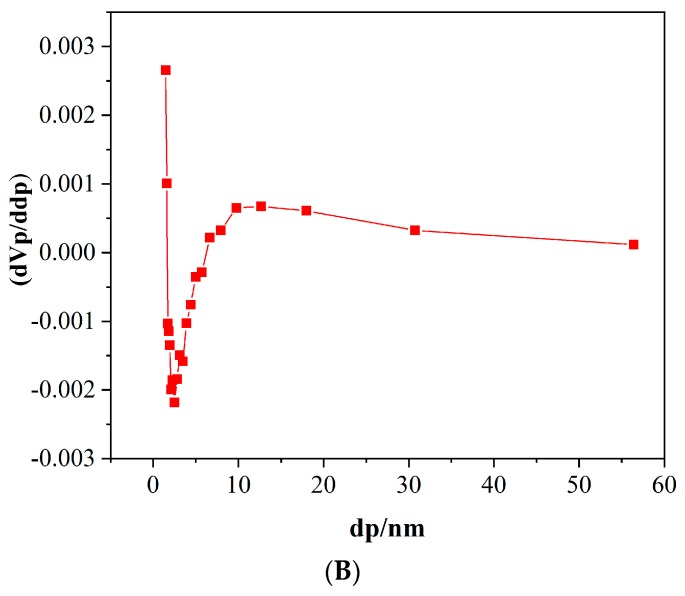
(**A**) N_2_ adsorption/desorption isotherms at −196 °C for Fe_3_O_4_/AC/CD/Alg polymer beads. (**B**) Pore size distribution of Fe_3_O_4_/AC/CD/Alg polymer beads.

Figure 6A showed the N_2_ adsorption and desorption isotherms for Fe_3_O_4_/AC/CD/Alg at −196 °C. The Brunauer–Emmett–Teller (BET) analysis was utilized for determining the surface area of the material. The BET specific surface area of was 8 m^2^ g^−1^, and Figure 6B showed the total pore volume was 0.02 cm^3^ g^−1^ with a mean pore diameter of 1.47 nm. This shows that the surface area of the material is larger than of other adsorbents [51]. The textural properties of the Fe_3_O_4_/AC/CD/Alg nanocomposite are shown in Table 2.

### 3.2. Effect of pH on Methylene Blue Adsorption

Figure S3A shows the effect of the dye solution pH range from 2 to 8 on the amount of dye adsorption capacity (initial dye concentration = 5 ppm, dosage Fe_3_O_4_/AC/CD/Alg polymer beads = 0.2 g/10 mL dye solution, agitation speed = 150 rpm, contact time = 90 min, room temperature). These dye sorption behaviors could be explained the following way: at low pH, the lower dye adsorption onto Fe_3_O_4_/AC/CD/Alg can be linked to the large amount of H^+^ ions present, which compete with the dye positive groups for the adsorption sites of the beads. With the increase of the pH of the solution (above 3), the active sites of the surface of the Fe_3_O_4_/AC/CD/Alg onto the hybrid beads were deprotonated and the competition for the adsorption sites between the dye cations and H^+^ decreased, which increased the amount of dye adsorbed [52]. Thus, the larger values found at pH above 3 are explained by electrostatic attraction forces between the immobilized negatively charged Fe_3_O_4_/AC/CD/Alg polymer beads and the positively charged dye ions. Similar results were observed for contact times of 60 min (Figure S3B) and 120 min (Figure S3C). For the dry powdered form of gel beads, the adsorption process increased rapidly and reached a maximum level of 98.6% and the optimum pH for the MB removal was found to be 6.

### 3.3. Effect of Adsorbent Amount

The effect of adsorbent amount on the adsorption efficiency of MB is illustrated in Figure S4A. The adsorbent dosage was varied from 0.04 to 0.5 g in 5 mg/L of MB dye at pH 6. The dye percentage removal by Fe_3_O_4_/AC/CD/Alg polymer gel beads increased from 84.76% to 99.43% and from 99.6% to 99.82% by dry Fe_3_O_4_/AC/CD/Alg polymer beads, as the adsorbent amount increased from 0.04 to 0.5 g/L. Figure S4B shows that the adsorption capacity diminished with an additional increase in Fe_3_O_4_/AC/CD/Alg polymer beads. Fe_3_O_4_/AC/CD/Alg dry polymer beads showed maximum adsorption capacity and high removal rate compared to gel polymer beads. This might be due to the enlarged surface area of the adsorbent and the availability of a larger amount of active adsorptive sites due to the increase in the quantity of dry polymer beads [53]. Thus, the 0.2 g amount of Fe_3_O_4_/AC/CD/Alg polymer gel beads and 0.06 g of dry beads were used for further study.

### 3.4. Effect of Initial Dye Concentration

**Figure 7:**
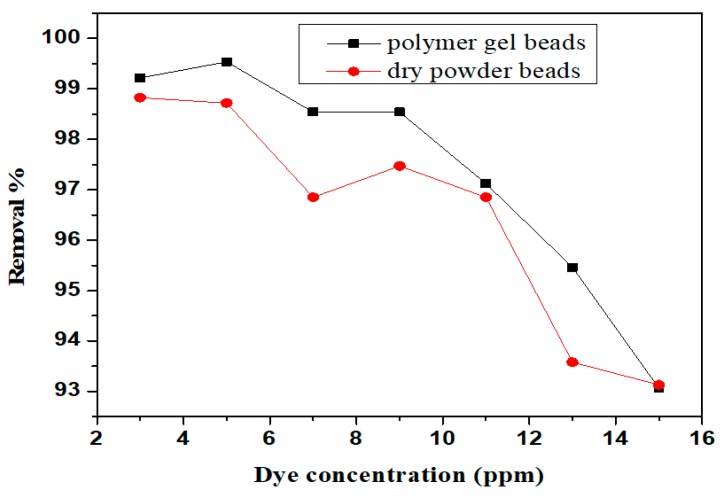
Effect of initial dye concentration on methylene blue (MB) adsorption by polymer gel beads and dry powder beads.

The amount of adsorbed dye was highly dependent on the initial concentration. To test such effect, the optimal amounts of gel beads (0.2 g) and dry powder beads (0.06 g) were agitated for 90 min. The obtained results are shown in Figure 7. The maximum removal was found at 5 ppm, with a maximum removal of 99.43% for gel beads, and 98.83% for dry beads, respectively, followed by a decrease. This can be explained by the adsorption sites saturation of the adsorbent surface, which indicated a possible formation of a monolayer of dye molecules at the interface with the adsorbent.

### 3.5. Effect of Contact Time on MB Adsorption

The adsorption capacity was determined as a function of time to evaluate the maximum time of contact during the adsorption process. Figure S5 showed the effect of contact time on the initial concentration of MB. The uptake of MB is fast at the initial stages and quickly comes near to equilibrium. This behavior could be explained as a large number of vacant surface sites were available for adsorption on the initial stage, and after a certain time, the remaining vacant surface sites were not easy to be occupied due to repulsive forces between solute molecules on solid and bulk phases [54]. Figure S5 clearly shows the equilibrium time of 90 min for removal MB by polymer gel beads and 50 min by dry powder beads. Dry beads moved fast towards equilibrium time, which indicated the possibility of the adsorption sites on the adsorbent surface to be early occupied by MB. Figure S5 plots are single, smooth and constant, leading to saturation, suggesting the possibility of formation of monolayer coverage of the dye [55].

### 3.6. Effect of Temperature

**Figure 8:**
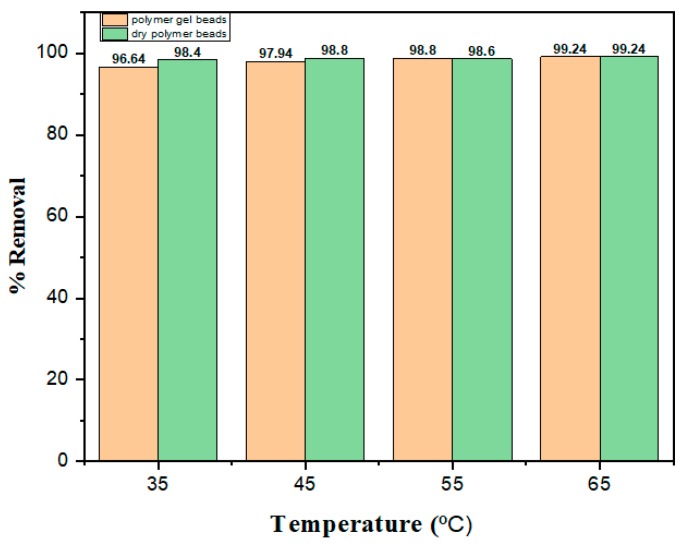
Effect of temperature on the removal of MB using polymer gel beads and dry powder beads (concentration of 5 mg L^−1^, pH 6, contact time of 90 min).

To determine the effect of temperature on adsorption, tests were carried out at four different temperatures, i.e., 35 °C, 45 °C, 55 °C and 65 °C, at an initial dye concentration of 5 mg L^−1^, pH 6 and contact time of 90 min. As shown in Figure 8, the adsorptive removal rate increased when the temperature was increased. It is known that a rise in temperature led to higher mobility of the dye molecules. The latter obtained enough energy to interact with the surface active sites. The uptake percent increased at earlier steps of adsorption and the removal amount reached to an approximate equilibrium for all temperature values. This was ascribed to the increase in the diffusion speed at higher temperature that allowed the MB molecules to quickly get into the inside of hydrogel beads, which led to an increase of the removal. This behavior confirms that the adsorption of dyes has an endothermic nature [56].

### 3.7. Adsorption Isotherms

**Figure 9:**
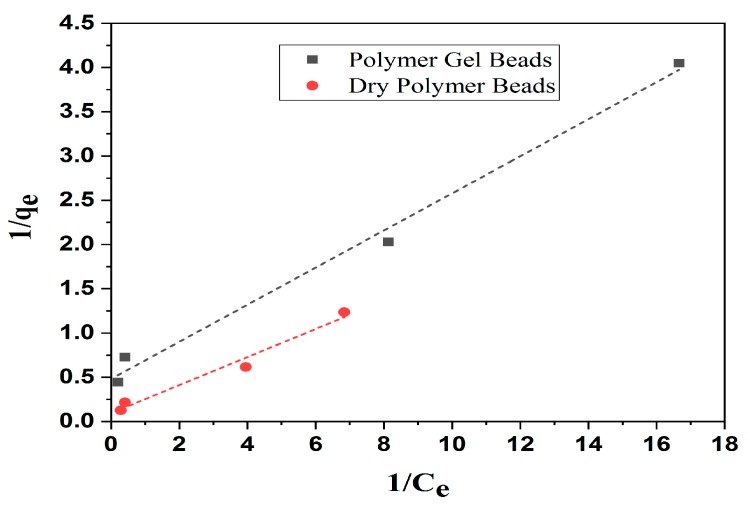
Langmuir adsorption isotherm fitting on data for MB adsorption on Fe_3_O_4_/AC/CD/Alg polymer gel beads and dry powder polymer beads.

Adsorption isotherm models have been often used to explain the adsorption processes and mechanisms. They describe the interaction between adsorbate and adsorbent, under propitious conditions. The obtained equilibrium data were fitted to several theoretical models, as Langmuir [57] and Freundlich [58], or empirical equations to interpret of the results, adsorption mechanism prediction, evaluation of parameters and the correlation coefficients were compared to check which model fitted better. Figure 9 shows that the adsorption capacity of Fe_3_O_4_/AC/CD/Alg polymer gel beads and dry powder beads for removal of MB. The Langmuir model is applicable for monolayer adsorption and this isotherm assumes that the adsorbent surface contains active sites with uniform monolayer formation energy. The linearized Langmuir isotherm model is represented as:

(5) $$\frac{1}{q_{e}}=\frac{1}{q_{m}}+\frac{1}{k_{L}q_{m}}\times \frac{1}{C_{e}},$$

where $q_{m}$ (mg/g) is the maximum adsorption amount on the adsorbent surface with complete monolayer coverage, *K_L_* (L/mg) is the Langmuir adsorption constant, $C_{e}$ (mg L^−1^) is the equilibrium concentration and $q_{e}$ (mg/g) is the amount adsorbed at equilibrium per adsorbent weight.

The main characteristics of the Langmuir adsorption can be expressed in terms of a constant (that is dimensionless), called separation factor or equilibrium parameter, used to predict if an adsorption system would be favorable or unfavorable. *R_L_* can be calculated by the equation below:

(6) $$R_{L}=\frac{1}{K_{L}C_{O}},$$

where $C_{O}$ (mg L^−1^) is the initial dye concentration. The $R_{L}$ values inform if the adsorption is irreversible ($R_{L}$ = 0), favorable (0 < $R_{L}$ < 1), linear (*R_L_* = 1) or unfavorable ($R_{L}$ > 1).

The multilayer adsorption heterogeneous surface energy is described by the Freundlich isotherm that can be linearized as:

(7) $$log\;q_{e}=log\;K_{F}+\frac{1}{n}\;log\;C_{e},$$

where *K_F_* (mg g^−1^) is the adsorption capacity and *n* is an empirical parameter that varies with the intensity and heterogeneity of the adsorption. The values of *1/n* and *K_F_* are calculated from the intercept and slope of the log $q_{e}$ versus *log*$C_{e}$ linear plot of Figure S6.

The adsorption parameters derived from the isotherm models are in Table 3.

The equilibrium data demonstrated that the Langmuir (R^2^ = 0.992) model showed a better fitting than the Freundlich model (R^2^ = 0.971), proving the surface homogeneity of the adsorbent and showing that the monolayer adsorption capacity ($q_{max}$) was 2.079 mg/g for polymer gel beads and 10.63 mg g^−1^ for dry powder beads. According to these results, the Fe_3_O_4_/AC/CD/Alg polymer nanocomposites can effectively be used for MB adsorption.

### 3.8. Adsorption Kinetics

**Figure 10:**
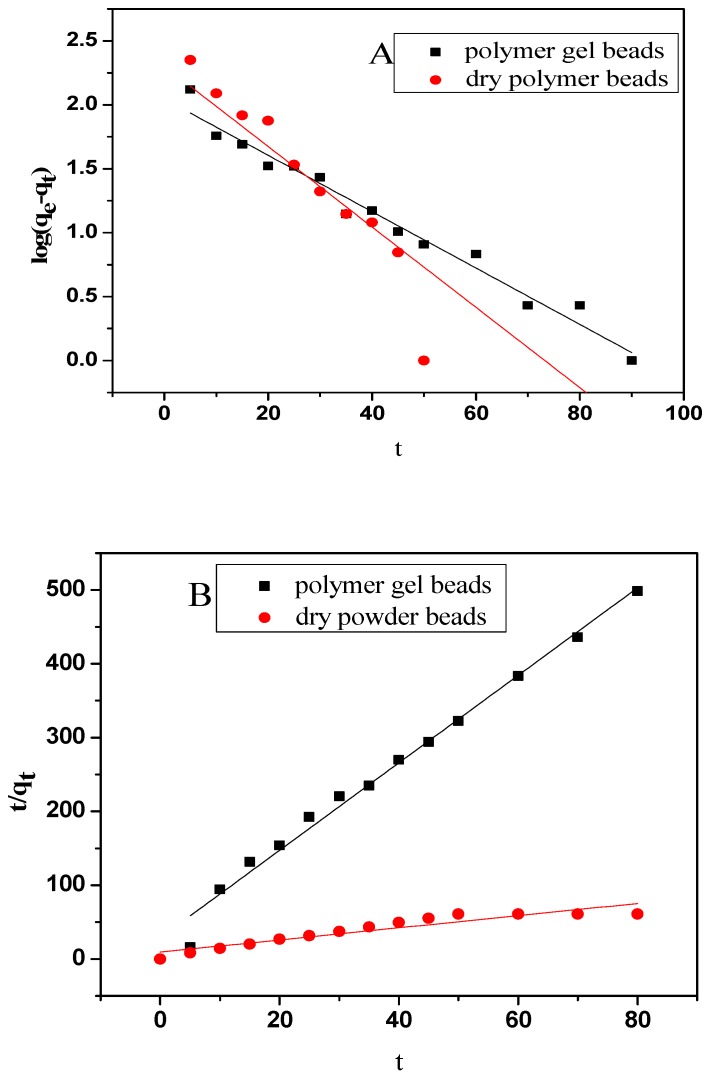
Adsorption of MB onto polymer gel beads and dry polymer beads fitted with the pseudo-first-order model (**A**) and the pseudo-second-order model (**B**).

The effect of contact time on MB removal by Fe_3_O_4_/AC/CD/Alg polymer beads was presented in Figure 10A,B). A good removal was found as the adsorption started and time approached 90 min. The MB adsorption kinetics was explained by predictive theoretical pseudo first order and pseudo second order models [59]. Adsorption kinetics was used to test the linearity between time and adsorption capacity. The usual models include the pseudo-first-order kinetic Equation (8) and the pseudo-second-order kinetic Equation (9) [60]. Pseudo first order kinetic model has the following equation:

(8) $$\log\;(q_{e}-q_{t})=\log q_{e}-\frac{k_{1}}{2.303}\times t,$$

where $q_{e}$ (mg/g) and $q_{t}$ (mg/g) are the adsorption capacities at equilibrium and at a given time *t* and k_1_ (min^−1^) is the rate constant, determined from the slope. Figure 10A shows log ($q_{e}-q_{t}$) versus *t* plot.

The pseudo second order kinetic model has this equation:

(9) $$\log\;(q_{e}-q_{t})\;=\log q_{e}-\frac{k_{1}}{2.303}\times t,$$

where $q_{e}$ and $\;q_{t}$ are the same as above, and *k*_2_ (g/mg min^−1^) is the rate constant. The values of $q_{e}\;\mathrm{and}\;k_{2}$ are calculated from the intercept and slope of the plot of *t*/$q_{t}$ versus *t* shown in Figure 10B. The pseudo second order kinetic model shows an excellent correlation coefficient (above 0.99) at room temperature. This showed that chemisorptions were the rate controlling step of MB adsorption onto Fe_3_O_4_/AC/CD/Alg polymer beads.

The pseudo first and second order rate constants and correlation coefficients are listed in Table 4.

The high correlation coefficient obtained for pseudo second order kinetics indicated that the model nicely represented the experimental data.

### 3.9. Thermodynamic Studies

The adsorption thermodynamic parameters, like enthalpy (Δ*H*°), entropy (Δ*S*°) and Gibbs free energy (Δ*G*°) change were calculated by the Equations (10)–(12), respectively [61].

(10) $$\Delta G{}^{\circ}=-RT\;\ln k_{c},$$

(11) $$\ln k_{d}=\frac{\Delta S{}^{\circ}}{R}-\frac{\Delta H{}^{\circ}}{RT},$$

(12) ΔG°=ΔH°−TΔS°,

where $\ln k_{d}$(L kg^−1^) is the distribution coefficient, Δ*G*° (kJ mol^−1^), Δ*H*°(kJ mol^−1^) and Δ*S*°(kJ mol^−1^) are the changes in Gibbs free energy, enthalpy and entropy, respectively, *T* is the temperature (in K). The adsorption capacity of the Fe_3_O_4_/AC/CD/Alg polymer composite beads increased with a temperature increase from 35 to 55 °C. Van’t Hoff Equation (11) related the distribution coefficient with ΔH° and Δ*S*° at constant temperature (1/*T*). The values of entropy and enthalpy were calculated from the intercepts and slopes of ln $k_{d}$ versus 1/*T* linear regression. Δ*H*° had a positive value suggesting the reaction to be endothermic, favoring the adsorption of the MB at higher temperature. The positive value of Δ*S*° indicated high randomness of adsorption, favoring the constancy of adsorption. The negative values of (Δ*G*°) confirmed the viability and spontaneity of the adsorption. Table 5 shows that an increase in temperature led to more efficient adsorption of the dye.

### 3.10. Desorption and Reusability Study of Fe_3_O_4_/AC/CD/Alg Polymer Beads

**Figure 11:**
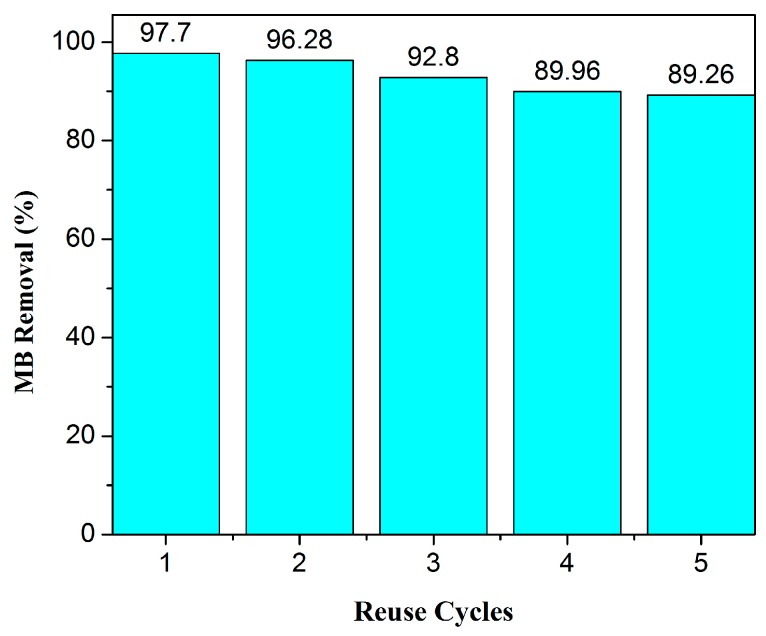
Reusability of Fe_3_O_4_/AC/CD/Alg polymer gel beads using 0.1 N HCl solutions up to five cycles of MB removal from the solution.

The reusability was tested up to five consecutive adsorption-desorption cycles and results are shown in Figure 11. In the fifth cycle, a mass loss caused by the acid used for regeneration led to a decrease in the maximum adsorption capacity.

### 3.11. Comparison with other Adsorbents

The adsorption capacity of MB on Fe_3_O_4_/AC/CD/Alg polymer beads was considerable, compared to other biological adsorbents found in literature (Table 6). The sodium alginate and CD are biodegradable and have a good prospect. The advantages of the method are the low manufacturing cost, good quality and easiness to synthesize the Fe_3_O_4_/AC/CD/Alg polymer beads.

## 4. Conclusions

In this work, novel Fe_3_O_4_/AC/CD/Alg polymer beads were successfully synthesized. Magnetic cross-linked AC/CD functionalized by Alg polymer was included, to generate effective active sites for the adsorption of the MB cationic dye from aqueous solutions. Magnetic nanoparticles in composites tended to form agglomerates to reduce the energy associated with the high surface area-to-volume ratio characteristic of nano-sized materials. To prevent aggregation of the magnetic nanoparticles, some protection approaches were created to stabilize the magnetic nanoparticles by coating with an organic (e.g., polymeric) or inorganic (e.g., activated charcoal) layer.

The effects of several parameters on the adsorption process (like pH, contact time temperature and initial dye concentration) were discussed. The versatility in surface modifications of magnetic nanoparticles offers advantages for diverse surface modifications. They could be easily functionalized using many different kinds of modification agents, including organic acids and polymers, based on their potential applications.

Magnetic separation shows advantages, like easy separation from solutions and possibility to treat a large amount of wastewater within a short time. Moreover, enhanced performances were achieved, due to cooperative interactions between the constituents.

Furthermore, a MB removal percentage of 99.53% at pH 6 was found, and the adsorbent could easily be regenerated and recycled, being reused up to five adsorption–desorption cycles, without any significant weight loss. The use of dry powder beads considerably enhanced the adsorption of MB, compared to gel beads, in terms of time and dosage.

In conclusion, this work opened a new path for effective elimination of cationic dyes from wastewater using an environmentally friendly biodegradable adsorbent. The Fe_3_O_4_/AC/CD/Alg polymer nanocomposite is a good adsorbent for large scale wastewater purification since it has high adsorption capacity and easy separation.

## Figures and Tables

**Table 1:** Crystallite size values calculated using the Debye–Scherrer formula.

Substance	Most Intense Peak (2θ, degree)	Most Intense Peak (θ, degree)	hkl	FWHM * of Most Intense Peak (β, Radians)	Size of the Particles (*D*, nm)
Fe_3_O_4_/AC	35.71	17.86	311	0.79	11.04
Fe_3_O_4_/AC/CD	30.41	15.20	220	3.11	2.71
Fe_3_O_4_/AC/CD/Alg	35.72	17.86	311	1.55	5.63

* Full width at half maximum height.

**Table 2:** Textural properties of the Fe_3_O_4_/AC/CD/Alg nanocomposite.

BET Analysis of the Fe_3_O_4_/AC/CD/AlgNanocomposite	
Specific surface area	8 m^2^ g^−1^
Total pore volume	0.02 cm^3^ g^−1^
Mean pore diameter	1.47 nm

**Table 3:** Equilibrium isotherm parameters of removal of MB by Fe_3_O_4_/AC/CD/Alg polymer gel beads and dry polymer beads.

Adsorbent	Langmuir				Freundlich		
	*K_L_* (L/mg)	$q_{m}\;(\mathrm{mg}/g)$	R^2^	*R_L_*	*K_F_*	*n*	R^2^
Fe_3_O_4_/AC/CD/Alg polymer gel beads	2.304	2.079	0.992	0.086	12.941	2.232	0.971
Dry polymer beads	0.595	10.638	0.976	0.336	17.458	1.597	0.963

**Table 4:** Kinetic parameters for the adsorption of MB onFe_3_O_4_/AC/CD/Alg polymer gel beads and dry polymer beads.

Adsorbent	Pseudo First Order			Pseudo Second Order		
	*k*_1_ (min^−1^)	$q_{e}\;(\mathrm{mg}/g)$	R^2^	*k*_2_ (g/mg min^−1^)	$q_{e}\;(\mathrm{mg}/g)$	R^2^
Fe_3_O_4_/AC/CD/Alg polymer gel beads	0.0483	99.083	0.980	1.243	0.168	0.987
Dry polymer beads	0.071	200.44	0.876	0.072	1.215	0.880

**Table 5:** Thermodynamic parameters for MB adsorption on Fe_3_O_4_/AC/CD/Alg polymer gel beads and dry powder beads.

Adsorbent	ΔH (KJ/mol)	ΔS (J/Kmol)	ΔG (KJ/mol)		
			308 K	318 K	328 K
Fe_3_O_4_/AC/CD/Alg polymer gel beads	15.77	56.80	−4.90	−5.591	−7.962
Dry polymer beads	0.024	92.867	−3.894	−5.062	−5.767

**Table 6:** Comparison of Fe_3_O_4_/AC/CD/Alg polymer gel beads with previously reported adsorbents.

Adsorbents	Adsorption Capacity (mg/g)	References
Walnut shell-activated carbon	3.53	[62]
Almond shell-activated carbon	1.33	[62]
Corncob derived activated carbon	0.84	[63]
Fir wood derived activated carbon	1.21	[64]
Alginate grafted polyacrylonitrile beads	3.51	[65]
NaOH-modified rejected tea	2.44	[66]
Fe_3_O_4_/AC/CD/Alg polymer beads	2.079	This study
Dry polymer beads	10.638	This study

## Supplementary Materials

The following are available online at https://www.mdpi.com/2079-4991/10/1/170/s1, Figure S1: (A) The chemical structure of Methylene Blue Dye, (B) Scheme outlining all the steps involved in the synthesis of Fe_3_O_4_ /AC, Fe_3_O_4_/AC/CD and Fe_3_O_4_/AC/CD/Alg nanocomposite from preparation to analysis, Figure S2: (A) Fe_3_O_4_/AC/CD/Alg polymer gel beads (B) image of Fe_3_O_4_/AC/CD/Alg nanocomposite attracted by a magnet (C) Before and after adsorption of MB solution by dry powder beads and (D) polymer gel beads, Figure S3: Effect of the dye solution pH range from 2 to 8 on the amount of dye adsorption capacity (initial dye concentration = 5 ppm, dosage Fe_3_O_4_/AC/CD/Alg polymer beads = 0.2 g/10 mL dye solution, agitation speed = 150 rpm, room temperature), with contact time = 90 min (A), 60 min (B) and 120 min (C), Figure S4: (A) Effect of adsorbent dosage on the adsorption of MB by polymer gel beads and dry powder beads (mass of catalyst = 0.04–0.5 g, pH= 6), (B) Effect of adsorbent dosage on the adsorption capacity and % removal of MB for Fe_3_O_4_/AC/CD/Alg polymer beads (initial dye concentration = 5 ppm, pH = 6, contact time = 90 min), Figure S5: Effect of contact time on MB adsorption by polymer gel beads and dry powder beads (initial MB concentration = 5 mg/L; adsorbent dose = 0.02 g; pH = 6), Figure S6: Fit of Freundlich isotherm on MB adsorption on Fe_3_O_4_/AC/CD/Alg polymer gel beads and dry powder polymer beads.
