# Supplementary material for: Cationic Dye Removal Using Novel Magnetic/Activated Charcoal/β-Cyclodextrin/Alginate Polymer Nanocomposite
Source: Nanomaterials (Basel). 2020 Jan 18;10(1):170. doi: 10.3390/nano10010170 (PMC7023446; doi:10.3390/nano10010170)
Supplement: Supplementary file 1 [file nanomaterials-10-00170-s001.pdf]

Supplementary Materials:

Cationic Dye Removal Using Novel Magnetic/Activated Charcoal/β-Cyclodextrin/Alginate Polymer Nanocomposite

Sushma Yadav [1], Anupama Asthana [1], Rupa Chakraborty [1], Bhawana Jain [1], Ajaya Kumar Singh [1,*], Sónia A. C. Carabineiro [2] and Md. Abu Bin Hassan Susan [3]

[1] Department of Chemistry, Govt. V.Y.T. PG Autonomous College, Durg 491001, India; sushmabhilai80@gmail.com (S.Y.); anurakeshbhilai@gmail.com (A.A.); roopachakraborty1991@gmail.com (R.C.); bhawanajain123@gmail.com (B.J.)

[2] Centro de QuímicaEstrutural, Instituto Superior Técnico, Universidade de Lisboa, Av. RoviscoPais 1, 1049-001 Lisboa, Portugal; sonia.carabineiro@tecnico.ulisboa.pt

[3] Department of Chemistry, University of Dhaka, Dhaka 1000, Bangladesh; susan@du.ac.bd

* Corresponding: ajayaksingh_au@yahoo.co.in; Tel.: +91-9406207572; Fax: +91-788-2211688

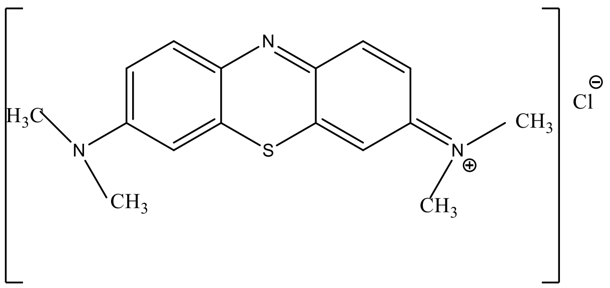

Figure S1. (A) The chemical structure of Methylene Blue Dye.

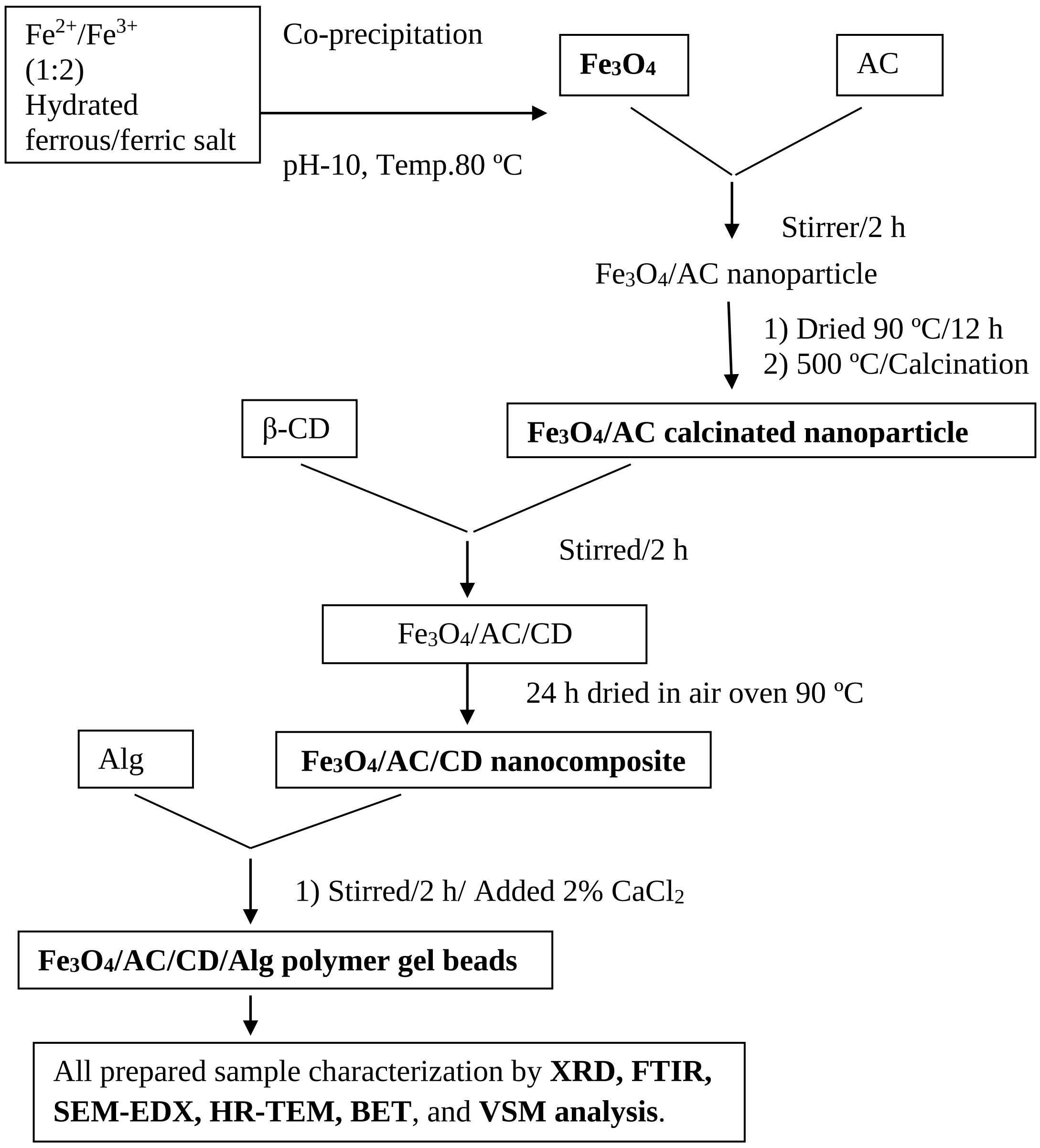

Figure S1. (**B**) Scheme outlining all the steps involved in the synthesis of Fe_3O_4 /AC, Fe_3O_4/AC/CD and Fe_3O_4/AC/CD/Alg nanocomposite from preparation to analysis.

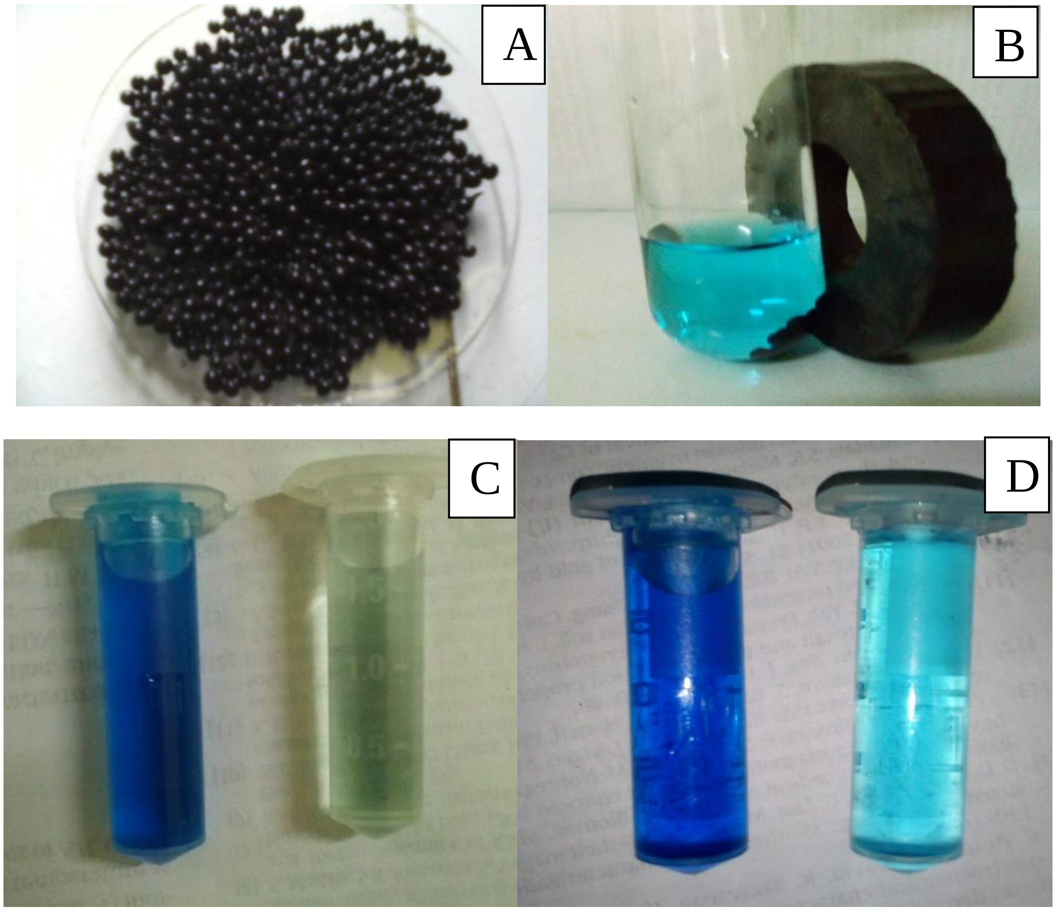

Figure S2. (**A**) Fe_3O_4/AC/CD/Alg polymer gel beads (**B**) image of Fe_3O_4/AC/CD/Alg nanocomposite attracted by a magnet (**C**) Before and after adsorption of MB solution by dry powder beads and (**D**) polymer gel beads.

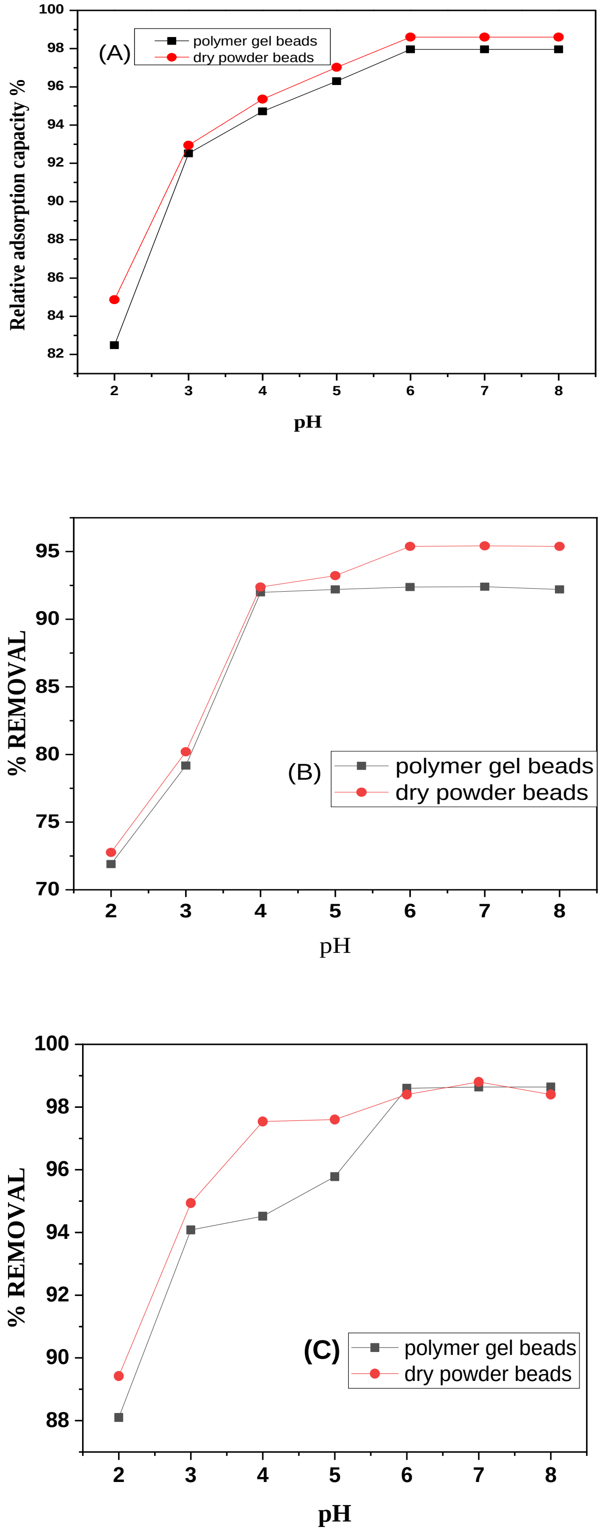

Figure S3. Effect of the dye solution pH range from 2 to 8 on the amount of dye adsorption capacity (initial dye concentration = 5 ppm, dosage Fe_3O_4/AC/CD/Alg polymer beads = 0.2 g/10ml dye solution, agitation speed = 150 rpm, room temperature), with contact time = 90 min (**A**), 60 min (**B**) and 120 min (**C**).

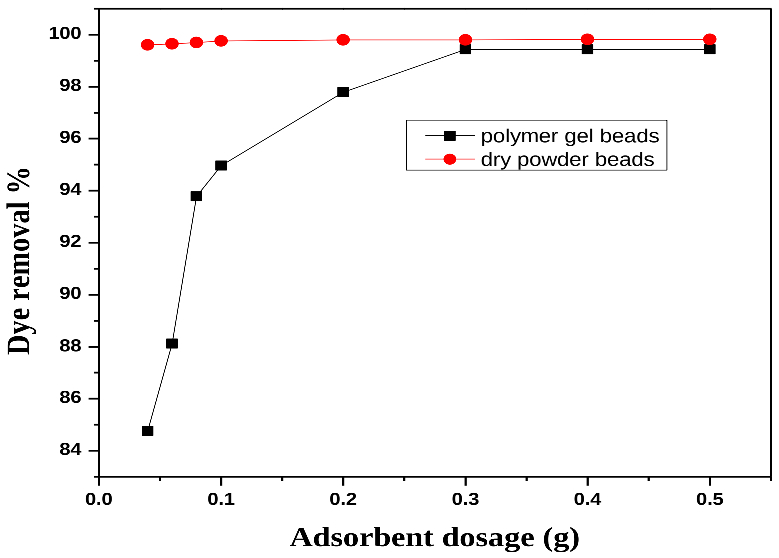

Figure S4. (**A**) Effect of adsorbent dosage on the adsorption of MB by polymer gel beads and dry powder beads (mass of catalyst= 0.04–0.5 g, pH= 6).

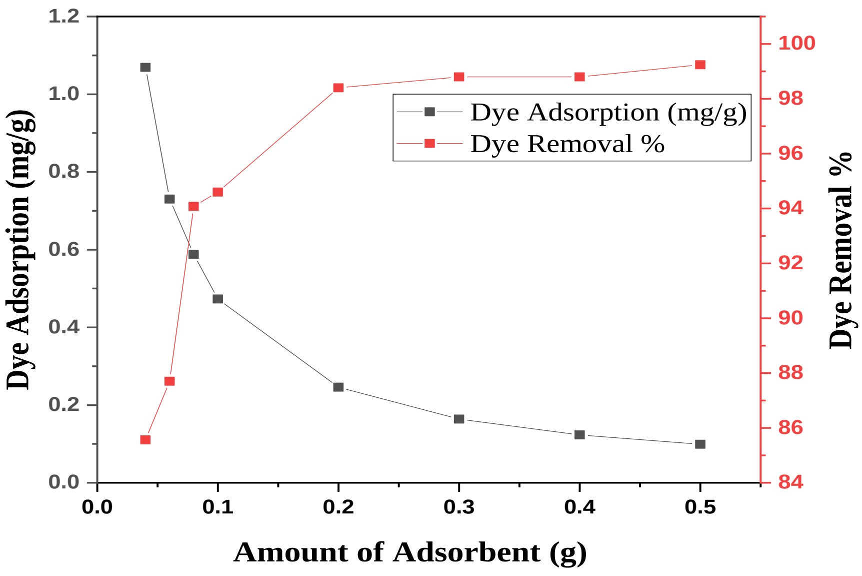

Figure S4. (**B**) Effect of adsorbent dosage on the adsorption capacity and % removal of MB for Fe_3O_4/AC/CD/Alg polymer beads (initial dye concentration = 5 ppm, pH = 6, contact time = 90 min).

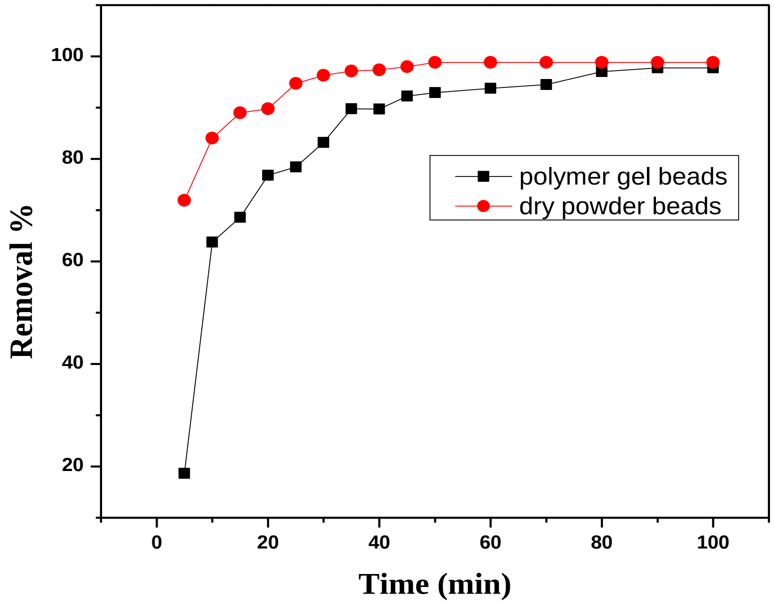

Figure S5. Effect of contact time on MB adsorption by polymer gel beads and dry powder beads (initial MB concentration = 5 mg/L; adsorbent dose = 0.02g; pH = 6).

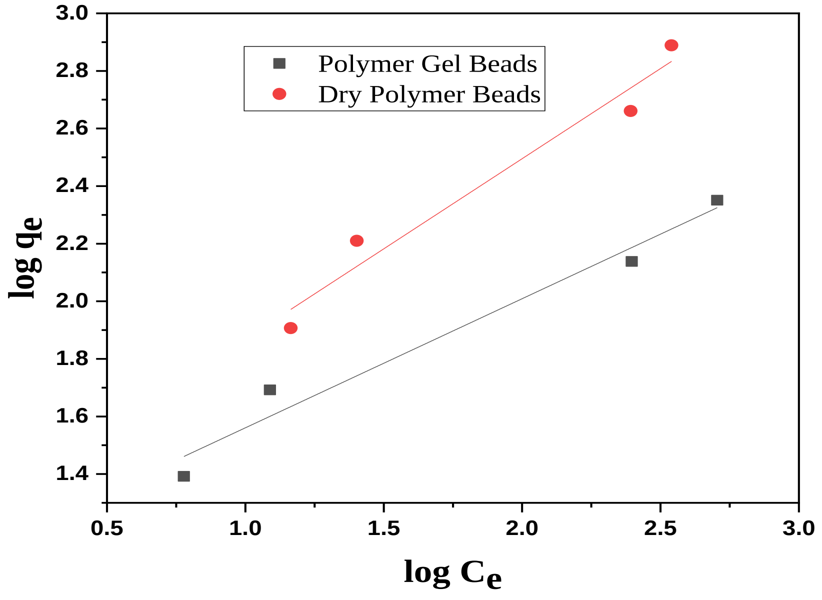

Figure S6. Fit of Freundlich isotherm on MB adsorption on Fe_3O_4/AC/CD/Alg polymer gel beads and dry powder polymer beads.
